# Inhibition of IL-1beta improves Glycaemia in a Mouse Model for Gestational Diabetes

**DOI:** 10.1038/s41598-020-59701-0

**Published:** 2020-02-20

**Authors:** Friederike Schulze, Josua Wehner, Denise V. Kratschmar, Valmir Makshana, Daniel T. Meier, Stéphanie P. Häuselmann, Elise Dalmas, Constanze Thienel, Erez Dror, Sophia J. Wiedemann, Shuyang Traub, Thierry M. Nordmann, Leila Rachid, Axel De Baat, Theresa V. Rohm, Cheng Zhao, Alex Odermatt, Marianne Böni-Schnetzler, Marc Y. Donath

**Affiliations:** 1 Clinic of Endocrinology, Diabetes and Metabolism, University Hospital Basel, Basel, Switzerland and Department of Biomedicine, University of Basel, Basel, Switzerland; 2 Division of Molecular and Systems Toxicology, Department of Pharmaceutical Sciences, University of Basel, Basel, Switzerland

**Keywords:** Gestational diabetes, Chronic inflammation

## Abstract

Gestational diabetes mellitus (GDM) is one of the most common diseases associated with pregnancy, however, the underlying mechanisms remain unclear. Based on the well documented role of inflammation in type 2 diabetes, the aim was to investigate the role of inflammation in GDM. We established a mouse model for GDM on the basis of its two major risk factors, obesity and aging. In these GDM mice, we observed increased Interleukin-1β (IL-1β) expression in the uterus and the placenta along with elevated circulating IL-1β concentrations compared to normoglycemic pregnant mice. Treatment with an anti-IL-1β antibody improved glucose-tolerance of GDM mice without apparent deleterious effects for the fetus. Finally, IL-1β antagonism showed a tendency for reduced plasma corticosterone concentrations, possibly explaining the metabolic improvement. We conclude that IL-1β is a causal driver of impaired glucose tolerance in GDM.

## Introduction

Gestational diabetes mellitus (GDM) is one of the most common pregnancy accompanying diseases. GDM is defined as glucose intolerance with the onset or first recognition during pregnancy^1^ and usually disappears after giving birth^2^. It was estimated that GDM affects 18.4 million pregnancies and 14% of all live births worldwide^2,3^. The incidence of GDM is associated with overweight and increased age of mothers, affecting 26% of pregnant women older than 45 years^3^ and 39% of pregnant women with a BMI ≥29 kg/m^2^ ^4^.

GDM is an independent risk factor for multiple complications including preeclampsia^5^, birth complications due to macrosomia, neonatal hypoglycaemia, jaundice^6^, and for life-long risk for obesity and type 2 diabetes mellitus (T2DM), the latter for both, the children and the mothers^7–9^.

However, so far, the pathogenesis of GDM is not well understood. During a normal pregnancy, maternal insulin resistance is increased^10,11^, probably to facilitate glucose transport to the growing fetus. This is accompanied by increased maternal insulin production^10,12^. GDM is characterized by even more pronounced insulin resistance, accompanied by an insufficient increase in insulin production to overcome insulin resistance^12,13^.

In addition, GDM is associated with an imbalance of various immunological processes occurring during pregnancy. Cytokines including interleukin-1β (IL-1β), interleukin-6 (IL-6), and tumor necrosis factor-α (TNF-α) as well as inflammatory markers such as C-reactive protein (CRP) are increased in the circulation of obese pregnant women and women with GDM^14–16^. Conversely, IL-1 receptor antagonist (IL-1Ra), an endogenous competitive inhibitor of IL-1 signaling, is decreased in plasma of women with GDM^17^. This indicates a particular relevance of the master cytokine IL-1β, which can induce IL-6, TNF-α and CRP^18,19^. Furthermore, obese pregnant women have more inflammatory macrophages and an altered natural killer cell profile in the placenta compared to lean pregnant women^20,21^.

IL-1β is well known for its role in T2DM. Obesity-induced insulin resistance is mediated through inflammatory factors, including TNF-α and IL-1β^22–24^. In obese subjects and patients with T2DM, IL-1β secretion, presumably from tissue-resident macrophages^25^, is increased in insulin-sensitive tissues^23^. It is also known that IL-1β is upregulated in pancreatic islets of patients with T2DM^26,27^. In rodents, it contributes to β-cell glucotoxicity and lipotoxicity, which lead to β-cell destruction and dedifferentiation^28–30^. In clinical trials, IL-1Ra improved glycaemia and insulin secretion in patients with T2DM^31,32^. Moreover, the CANTOS trial involving 10’061 patients showed that IL-1β antagonism with canakinumab, a neutralizing anti-IL-1β antibody, is effective in reducing the glycated hemoglobin (HbA1c) during the first 6 months of treatment in patients with a history of myocardial infarction^33,34^. After 6 months, the glucose lowering effect remained visible only in the non-diabetic population, while in patients with diabetes the effect was lost due to change in diabetes medication. A recent meta-analysis of all 2921 reported cases with T2DM undergoing anti-IL-1 treatment demonstrated a significant (p < 0.05) reduction in HbA1c^32^.

Insulin resistance is partly mediated by steroid hormones such as cortisol, progesterone and estradiol, of which serum concentrations are increased during pregnancy and even further increased in patients with GDM^35,36^ The placenta is a production tissue of these steroid hormones and other insulin resistance-inducing hormones^35,37^. Interestingly, IL-1β has been shown to stimulate the production of various steroid hormones: it stimulates the production of progesterone from a human placental cell line^38^ and bovine granulosa cells^39^. In rodents, IL-1β increases serum corticosterone by stimulation of the hypothalamus, pituitary, adrenal (HPA) axis^40–43^, resulting in increased corticosteroid synthesis. Conversely, IL-1Ra inhibits stress-induced corticosterone production^44^, which underlines the link between IL-1 and steroid production. Similarly, in humans, administration of IL-1Ra decreases serum cortisol of healthy^45^ and obese individuals^46^.

Since IL-1β antagonism improves β-cell function^31^ in subjects with type 2 diabetes and insulin sensitivity in obese individuals^47^, and since IL-1β is increased in women with GDM^14,36^, we aimed to study the effect of IL-1β antagonism in this condition. Therefore, we established a mouse model of GDM and tested the effect of blocking IL-1β in comparison to vehicle-treated pregnant and non-pregnant mice.

## Materials and Methods

### Mice

All mouse experiments were approved by the cantonal authorities of Basel Stadt, Switzerland and research was conducted in accordance with the guidelines for the care and use of animals of the cantonal authorities of Basel Stadt, Switzerland. Naïve female C57BL/6N mice were either purchased from Charles River (Sulzfeld, Germany) or originated from our own in-house breeding. Naïve female *Lyz2*-Cre-specific IL-1β knock out mice (*Il1b*^fl/fl^*Lyz2*-Cre) and Cre-negative littermate controls (WT) originated from our in-house breeding^48^. All comparisons within the groups are comparisons between littermate mice.

### Timed-mating

**Figure 1:**
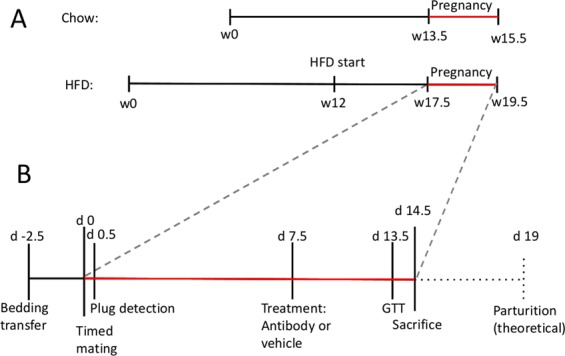
Schematic representation of the experimental timeline. w = week of age; d = day of pregnancy. (**A**) Timeline of lean mice and obese healthy and obese gestational diabetes mellitus mice. (**B**) Timeline of all three groups during pregnancy.

In order to align menstrual cycles and induce estrus in female mice, used bedding material from male mice was transferred into female study cages. 2.5 days after cycle synchronization, females were transferred into the cage of male breeders and returned to their own cage after 24 hrs. The day of plug detection was regarded as day 0.5 of pregnancy (Fig. 1B). Unfortunately, plug detection combined with weight analysis turned out to be often unspecific in predicting pregnancy, imposing us to use a large number of mice (total number of mice for the study was 346). At timed-mating, mice were on average 13.5 weeks old (chow-fed C57BL/6N mice and high-fat diet (HFD) -fed *Il1b*^fl/fl^*Lyz2*-Cre mice and their wild type controls) or 17.5 weeks old (HFD-fed C57BL/6N mice) (Fig. 1A).

### Anti-IL-1β treatment and diet

If indicated, mice received one single subcutaneous (s.c.) bolus injection containing 10 µg/g body weight of a murine anti-IL-1β antibody (BSUR05, with the same specificity as canakinumab; kindly provided by Novartis, Switzerland) used in previous studies^49^, or vehicle on day 7.5 of pregnancy.

If indicated, mice were fed HFD with 60 kJ% fat and 21 kJ% carbohydrates (Lard; EF D12492; ssniff Spezialitäten, Soest, Germany) starting at the age of 12 weeks, all other mice received normal rodent-chow diet (3436, Provimi Kliba, Kaiseraugst, Switzerland).

### Experimental groups

There were 168 chow-fed mice in 10 independent cohorts. We called these cohorts “lean” mice. There were 74 HFD-fed mice in 5 independent cohorts in which pregnant mice developed gestational diabetes. We called these cohorts oGDM. There were 61 HFD-fed mice in 4 independent cohorts in which pregnant mice did not develop gestational diabetes. We called these cohorts oGH. There were 43 HFD-fed *Il1b*^fl/fl^*Lyz2*-Cre mice and wild type controls in 3 independent cohorts.

### Environmental conditions

All mice had free access to food and water and were housed in a 12-hour light and dark cycle with the light phase during the day. Individually ventilated cages (Greenline-Tecniplast) contained 2–5 animals, environmental enrichment, ABEDD classic 4604 as bedding material and were kept in a specific pathogen free facility at 25 °C. Weight and health status was checked on a weekly basis.

### Glucose-tolerance testing

Glucose-tolerance testing (GTT) was performed on day 13.5 of pregnancy. For GTT, mice were fasted for 6 hours starting in the morning. Fasted mice received an s.c. injection of 2 g glucose per kg of body weight. Blood glucose was measured twice per time point prior to, and 15, 30, 60, 90, and 120 minutes after glucose application, each with a drop of blood from the tail vein, using FreeStyle Lite glucose meters (Abbott AG, Baar, Switzerland). At the first three time points, additional blood samples were collected from the tail vein into tubes containing EDTA for later measurement of plasma insulin with the MSD Mouse/Rat Insulin Kit (Meso Scale Diagnostics, Rockville MD, USA) according to manufacturer’s instructions.

### Sacrifice of mice

Mice were sacrificed in the afternoon of day 14.5 of pregnancy. Serum and tissue for IL-1β and steroid hormone measurements and gene expression analyses were obtained at sacrifice.

### Serum preparation

Serum was obtained by allowing fresh blood obtained by cardiac puncture to stand for 30 minutes at room temperature, followed by centrifugation at 10,000 rcf for 20 minutes.

### IL-1β measurements of serum and organ extracts

Serum-IL-1β measurements were performed with the MSD mouse IL-1β Kit (K152QPD; Meso Scale Diagnostics) according to the “alternative protocol 2” of the manufacturer’s instructions.

For tissue preparations, previously weighed pieces of tissue were homogenized with 5 mm stainless steel beads (69989; Qiagen, Maryland, USA) in a TissueLyser (85300; Qiagen) in protein lysis buffer containing 20 mM Tris buffer pH 7.5, 1% Triton X-100, 150 mM NaCl, 10% glycerol, 10% cOmplete Protease Inhibitor Cocktail (Roche, Switzerland), 10 mM Na_3_VO_4_, 100 mM NaF, 5 mM PMSF and 5 mM EDTA. The resulting homogenates were centrifuged two times at 10,000 rcf for 10 min at 4 °C to pellet insoluble material and the clear protein supernatant was collected. IL-1β concentration in supernatants was measured with the MSD mouse IL-1β Kit according to the “alternative protocol 2” of the manufacturer’s instructions. The detected IL-1β concentration was normalized to the weight of the processed tissue.

### IL-1Ra measurements of serum

Serum-IL-1Ra measurements were performed with the mouse IL-1ra Quantikine ELISA Kit (MRA00, Biotechne) according to the manufacturer’s instructions.

### Serum steroid hormone measurements

Serum steroid hormone levels [corticosterone, 11-dehydrocorticosterone, 11-deoxycorticosterone, aldosterone, androstenedione, testosterone and progesterone] were determined as described previously with minor adaptations^50^. Briefly, for solid-phase extraction (SPE), each serum sample (100 µL) was mixed with protein precipitation solution (100 µL, 0.8 M zinc sulphate in water/methanol; 50/50, v/v) that contained (33 nM) deuterium-labeled aldosterone (D7), corticosterone (D8), androstenedione (D7) and testosterone (D2) as internal standard. Prior SPE, all samples were diluted to a final volume of 1 mL with water and incubated in a thermoshaker (10 min at 4 °C, 1300 rpm). Following incubation, samples were centrifuged (10 min at 16,000 rcf at 4 °C), and supernatants (950 µL) were transferred to Oasis HBL SPE (1 cc) cartridges (Waters, Milford, MA, USA), preconditioned with methanol and water (3 × 1 mL each). Samples were washed with water (1 mL) and methanol/water (2 × 1 mL, 10/90, v/v). Steroids were eluted with methanol (2 × 0,75 mL), evaporated to dryness (3 hours, 35 °C) and reconstituted in methanol (25 µL, 10 min, 4 °C, 1300 rpm). Steroid content was analyzed by ultra-high pressure liquid chromatography-tandem mass spectrometry (UPLC-MS/MS) using an Agilent 1290 UPLC coupled to an Agilent 6490 triple quadrupole mass spectrometer equipped with a jet-stream electrospray ionization interface (Agilent Technologies, Santa Clara, CA, USA). Analyte separation was achieved using a reverse-phase column (1.7 µm, 2.1 mm × 150 mm; Acquity UPLC BEH C18; Waters). Data acquisition and quantitative analysis were performed by MassHunter (Version B.07.01, Agilent Technologies).

### Ribonucleic acid (RNA) extraction and quantitative PCR (qPCR)

Pieces of tissue (20–30 µg) were homogenized with 5 mm stainless steel beads (69989; Qiagen) in a TissueLyser (85300; Qiagen) in 350 µl lysis buffer of the RNA extraction kit and total RNA was isolated using the NucleoSpin RNA II Kit (Macherey Nagel, Düren, Germany) according to the manufacturer’s instructions. Complementary deoxyribonucleic acid was prepared using the GoScript Reverse Transcriptase (A5003, Promega, Catalys, Switzerland) and used for Taqman qPCR or Sybr Green qPCR on a ViiA 7 real-time PCR system (Thermo Fischer Scientific, USA). For Taqman qPCR we used GoTaq polymerase mixes (A6102, GoTaq Probe qPCR Master mix, Promega, Dübendorf, Switzerland) and the following ABI Taqman probes (Thermo Fisher Scientific, Reinach, Switzerland): *Il1b*: Mm00434228, *18s*: Hs99999901_s1. For Sybr Green qPCR we used GoTaq qPCR Master Mix (A6002, Promega, Catalys) and the following primers: *18S* fw 5′-GGGAGCCTGAGAAACGGC-3′ and rev 5′-GGGTCGGGAGTGGGTAATTT-3′, *Cyp11b2* fw 5′-CGTGGCCTGAGACGTGGTGT-3′ and rev 5′-CATCCATGGTAAGGCTCCCACGA-3′, *NK1*.*1* (Gene name: *Klrb1c*) fw 5′-GCTGTGCTGGGCTCATCCT-3′ and rev 5′-TTGATGGTTTTTGTACTAAGACTCGCA-3′, *Cd74* fw 5′-CCCAGGACCATGTGATGCAT-3′ and rev 5′-CTTAAGATGCTTCAGATTCTCT-3′, *Cd68* fw 5′-GCAGCACAGTGGACATTCAT-3′ and rev 5′-AGAGAAACATGGCCCGAAGT-3′, *Adgre* fw 5′-GCCCAGGAGTGGAATGTCAA-3′ and rev 5′-CAGACACTCATCAACATCTGCG-3′.

### Statistics

Data are expressed as means (SEM). The following statistical tests were performed where appropriate: Two-way ANOVA followed by Sidak’s multiple comparison analysis, Mann-Whitney U test, Dunn’s Kruskal-Wallis multiple comparisons. Tests as stated in the figure legends were used for comparison of groups and p < 0.05 were considered significant. Data analysis was performed using GraphPad Prism v7.0d software.

### Ethics

All animal experiments were approved by the cantonal authorities of Basel, Switzerland (license number 2695_28261).

## Results

### Mouse model for gestational diabetes mellitus

To establish a rodent model for GDM, we fed pregnant mice of different ages with standard chow or HFD and categorized them according to their glucose tolerance.

**Figure 2:**
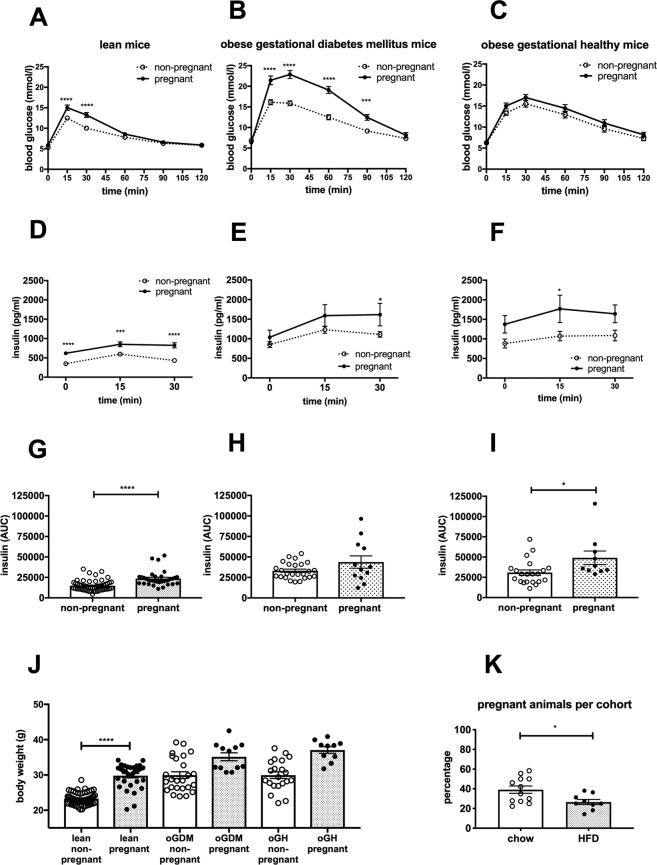
Mouse model for gestational diabetes mellitus. Concentration of (**A**–**C**) plasma glucose and (**D**–**I**) insulin during a s.c. GTT. (**A**,**D**,**G**); lean mice (non-pregnant n = 62, pregnant n = 35), (**B**,**E**,**H**); oGDM mice (non-pregnant n = 25, pregnant n = 12), (**C**,**F**,**I**); oGH mice (non-pregnant n = 21, pregnant n = 10). (**J**) Body weight was assessed on the day of GTT. (**K**) Percentage of mice per cohort becoming pregnant after timed-mating. *P < 0.05, **P < 0.01, ***P < 0.001, ****P < 0.0001 ((**A**–**F**) Two-way ANOVA followed by Sidak’s multiple comparison analysis, (**G**–**I**,**K**) Mann-Whitney U test, (**J**) Dunn’s Kruskal-Wallis multiple comparisons).

Glucose clearance in chow-fed, hereafter called “lean”, pregnant mice with the average mating age of 13.5 weeks was delayed compared to non-pregnant controls (Fig. 2A) despite (insufficiently) increased insulin secretion (Fig. 2D,G).

To induce GDM, we fed older mice (12 weeks of age) an HFD for 8 weeks before mating (average age at mating: 17.5 weeks) and during pregnancy, thereby applying two major risk factors for GDM. We then divided the HFD-fed cohorts depending on the development of impaired glucose tolerance. Similar to the frequencies observed in human obese pregnancies (39%), we saw a marked impairment of glucose tolerance in five of nine (55%) HFD-fed cohorts (average number of animals per cohort: 17). Glucose tolerance was markedly impaired, with a more than 20% higher rise in blood glucose in pregnant mice compared to non-pregnant controls (average AUC impairment by pregnancy: 37.25%, 99% CI 6.57%) (Fig. 2B). Plasma insulin was significantly (P < 0.05) increased only 30 min after the glucose bolus and insulin AUC was not different compared to non-pregnant controls (Fig. 2E,H). These HFD-fed cohorts are hereinafter referred to as “obese gestational diabetes mellitus” (oGDM) mice. In contrast, in four HFD-fed cohorts glucose tolerance was not impaired in pregnant mice compared to the non-pregnant controls (average AUC impairment by pregnancy: 9.07%, 99% CI 10.00%) (Fig. 2C) probably due to a significant (P < 0.05) increase in insulin secretion (Fig. 2F,I). These cohorts are herein referred to as “obese gestation healthy” (oGH).

As expected, pregnancy increased body weight in all three models by approximately 20%, although HFD feeding masked some of this effect (Fig. 2J). Importantly, body weight in pregnant oGDM and oGH mice was comparable.

An interesting side observation was that fertility was significantly (P < 0.05) lower in HFD-fed mice compared to lean mice (Fig. 2K).

### IL-1β is increased in pregnant mice

**Figure 3:**
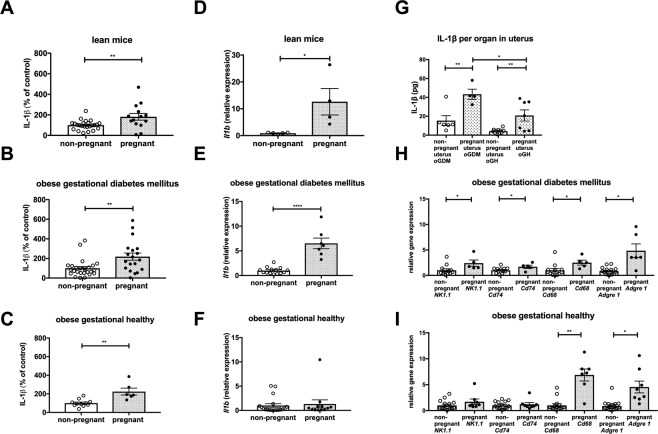
IL-1β is increased in pregnant mice. IL-1β normalized to average of non-pregnant mice measured in afternoon serum of (**A**) lean (non-pregnant n = 24, pregnant n = 14), (**B**) oGDM (non-pregnant n = 27, pregnant n = 19), (**C**) oGH (non-pregnant n = 11, pregnant n = 6). *Il1b* gene expression measured in uterine tissue from (**D**) lean (non-pregnant n = 5, pregnant n = 4), (**E**) oGDM (non-pregnant n = 14, pregnant n = 7), (**F**) oGH (non-pregnant n = 19, pregnant n = 12) mice. (**G**) IL-1β protein measured in uterine tissue of oGDM (non-pregnant n = 6, pregnant n = 4) and oGH (non-pregnant n = 11, pregnant n = 7) mice. Relative gene expression of immune cell markers measured in uterine tissue of (**H**) oGDM (non-pregnant n = 12, pregnant n = 5) and (**I**) oGH (non-pregnant n = 15, pregnant n = 8) mice. *P < 0.05, **P < 0.01, ****P < 0.0001 ((**A**–**I**) Mann-Whitney U test).

We then tested the hypothesis that IL-1β may impair glucose tolerance during pregnancy. First, we measured serum IL-1β in pregnant mice compared to their respective non-pregnant controls. IL-1β was elevated in all three pregnancy models (Fig. 3A–C) with a doubling of IL-1β levels in all three groups.

To track the source of the increase in circulating IL-1β, we measured IL-1β in various tissues relevant to metabolism and pregnancy. There was no difference in *Il1b* gene expression and protein level in subcutaneous or gonadal fat tissue, nor in spleen, liver or pancreatic islets between pregnant mice and non-pregnant controls (data not shown). In the uterus, however, *Il1b* gene expression was increased in pregnant lean and oGDM mice but not in pregnant oGH mice compared to their corresponding non-pregnant control (Fig. 3D–F). To test if this increase in gene expression translates to protein level, we also measured IL-1β content per uterus. The total amount of IL-1β per organ was higher in pregnant oGMD and oGH mice compared to corresponding non-pregnant controls, but this increase was lower in oGH (Fig. 3G). We also measured IL-1β protein content of placentae in oGDM and oGH mice, which was similar to that of the uterus in pregnant mice (Supplement Fig. S1A). This indicates that the uterus with the placentae is a probable source of increased IL-1β during pregnancy.

To substantiate an increase in biologically active IL-1β, we measured the expression of immune cell markers in the uterus. Compared to non-pregnant controls, oGDM mice had increased gene expression of immune cell markers, such as *NK1*.*1* for natural killer cells, *Cd74* for dendritic cells and *Cd68* and *Adgre* for macrophages (Fig. 3H). In pregnant oGH mice, only uterine gene expression of *Cd68* and *Adgre* but not of *NK1*.*1* and *Cd74* was increased (Fig. 3I).

### IL-1β antagonism improves glycaemia in GDM

**Figure 4:**
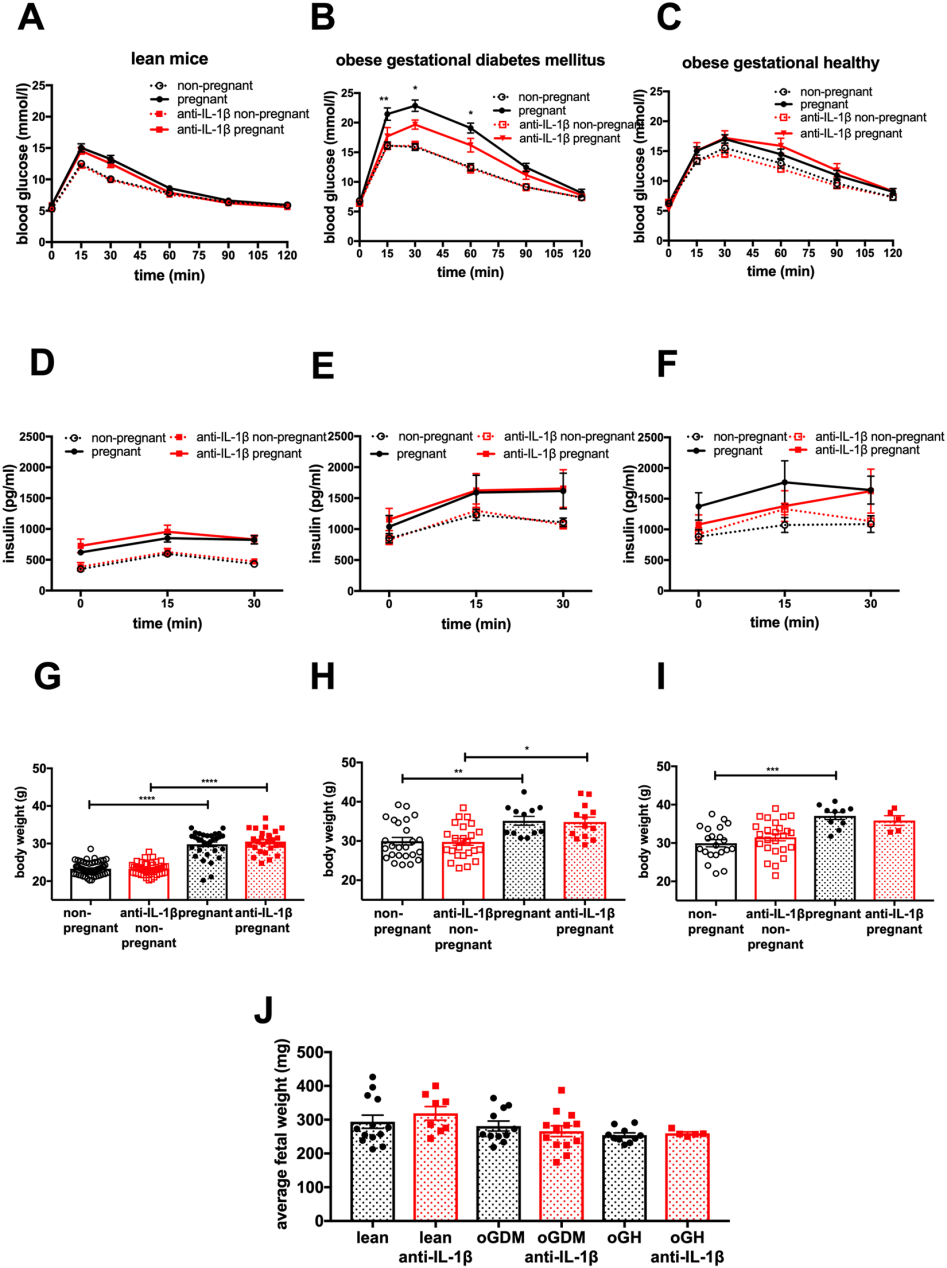
IL-1β antagonism improves glycaemia in gestational diabetes mellitus. Concentration of (**A**–**C**) plasma glucose and (**D**–**F**) insulin during a s.c. GTT in (**A**,**D**) lean (non-pregnant n = 62, anti-IL-1β treated non-pregnant n = 43, pregnant n = 35, anti-IL-1β treated pregnant n = 28), (**B**,**E**) oGDM (non-pregnant n = 25, anti-IL-1β treated non-pregnant n = 24, pregnant n = 12, anti-IL-1β treated pregnant n = 13) and (**C**,**F**) oGH (non-pregnant n = 21, anti-IL-1β treated non-pregnant n = 25, pregnant n = 10, anti-IL-1β treated pregnant n = 5) mice. Body weight was assessed in (**G**) lean, (**H**) oGDM and (**I**) oGH on the day of GTT. (**J**) Average fetal weight per pregnant mouse was assessed one day after GTT in pregnant lean (pregnant n = 13, anti-IL-1β treated pregnant n = 8), oGDM (pregnant n = 12, anti-IL-1β treated pregnant n = 13) and oGH (pregnant n = 10, anti-IL-1β treated pregnant n = 5) mice. *P < 0.05, **P < 0.01, ***P < 0.001, ****P < 0.0001 ((**A**–**F**) Two-way ANOVA followed by Sidak’s multiple comparison analysis (only significance of pregnant vs. pregnant anti-IL-1β treated represented), (**G**–**J**) Dunn’s Kruskal-Wallis multiple comparisons).

To test if IL-1β has a causal role in the pregnancy-associated impairment of glucose tolerance, we injected a neutralizing anti-IL-1β antibody (anti-IL-1β) into pregnant mice on day 7.5 of pregnancy and corresponding non-pregnant controls to block IL-1β action (Fig. 1B). Of note, the injections occurred before the pregnancy could be verified, forcing us to increase the number of experiments but with the benefit of better randomization. In non-pregnant mice as well as in pregnant lean and pregnant oGH mice, injection of anti-IL-β had no effect on glucose tolerance or basal and glucose-stimulated insulin secretion (Fig. 4A,C,D,F). In contrast, in pregnant oGDM mice, injection of anti-IL-1β significantly (P < 0.01) improved glucose tolerance (Fig. 4B), while basal and glucose-stimulated insulin secretion did not differ (Fig. 4E), pointing to changes in insulin sensitivity. Anti-IL-1β injections did not influence maternal or fetal body weight in either of the three pregnancy models (Fig. 4G–J). To investigate a potential counter-regulatory mechanism in the IL-1 pathway during GDM, we measured circulating IL-1Ra in oGDM and oGH mice. IL-1Ra was comparable between anti-IL-1β-treated or not pregnant mice and their respective non-pregnant controls (Supplement Fig. S1B,C).

To test the involvement of the myeloid compartment as a possible source of IL-1β in GDM we used myeloid cell-specific IL-1β knock out mice. In contrast to oGDM mice injected with anti-IL-1β, HFD-fed pregnant *Il1b*^fl/fl^*Lyz2*-Cre knock out mice were not protected from impaired glucose tolerance (Supplement Fig. S1D,E).

### Steroid hormones

To test whether IL-β regulates adrenal steroidogenesis and thereby possibly affects insulin resistance during pregnancy, we measured serum concentrations of a panel of steroid hormones in lean and oGDM mice and their non-pregnant controls treated with anti-IL-β or vehicle.

**Figure 5:**
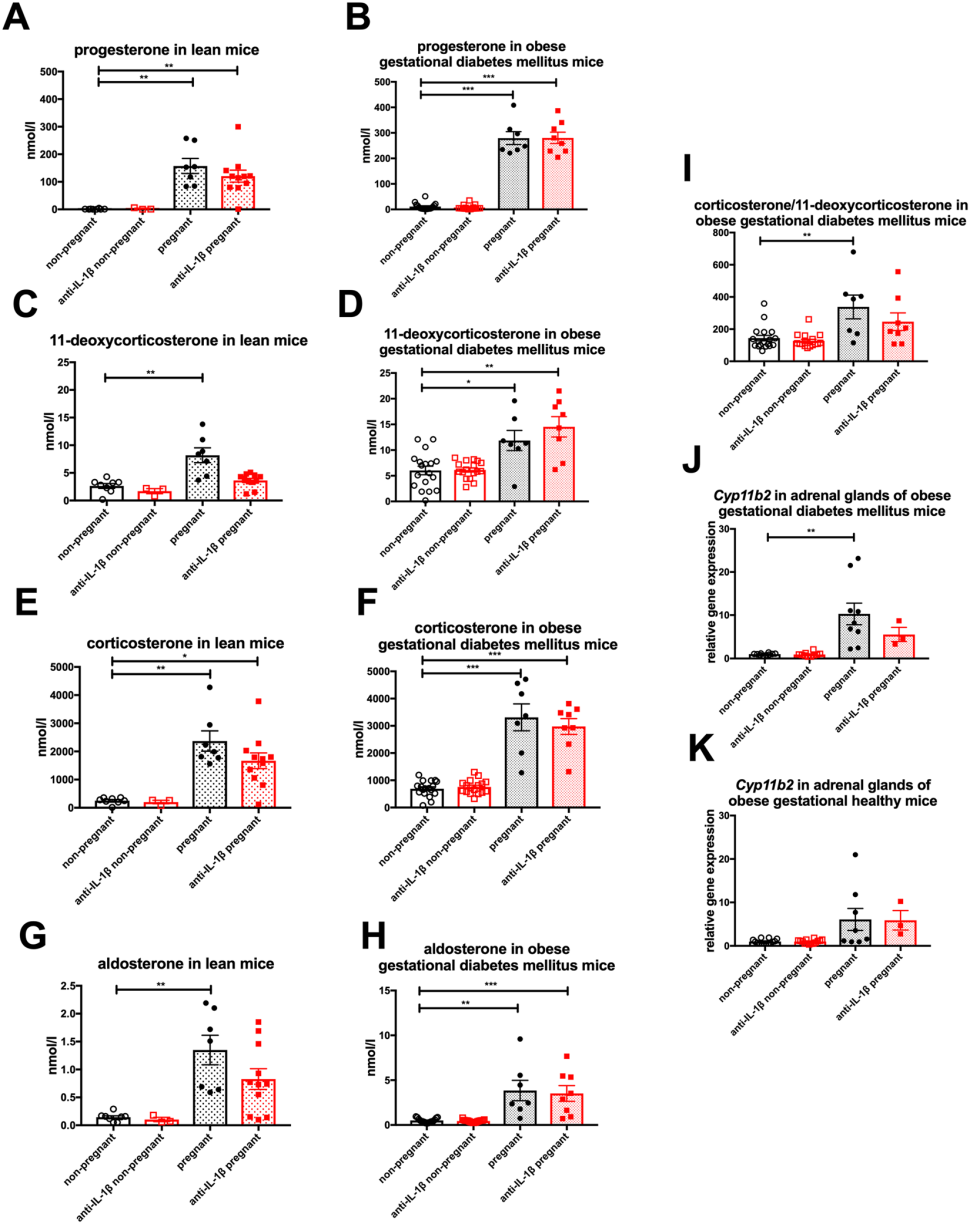
Changes in steroid hormones in gestational diabetes mellitus. (**A**) Progesterone, (**C**) 11-deoxycorticosterone and (**E**) corticosterone (**G**) aldosterone was measured in serum of non-pregnant (n = 8), anti-IL-1β treated non-pregnant (n = 3), pregnant (n = 7) and anti-IL-1β treated pregnant (n = 11) lean mice. (**B**) Progesterone, (**D**) 11-deoxycorticosterone, (**F**) corticosterone (**H**) aldosterone was measured in serum of non-pregnant (n = 18), anti-IL-1β treated non-pregnant (n = 17), pregnant (n = 7) and anti-IL-1β treated pregnant (n = 8) oGDM mice. (**I**) Corticosterone/11-deoxycorticosterone ratio was calculated from the measurements of (**D**,**F**). Gene expression of *Cyp11b2* was measured in adrenal tissue of (**J**) oGDM (non-pregnant n = 10, anti-IL-1β treated non-pregnant n = 10, pregnant n = 8, anti-IL-1β treated pregnant n = 3) and (**K**) oGH (non-pregnant n = 14, anti-IL-1β treated non-pregnant n = 14, pregnant n = 8, anti-IL-1β treated pregnant n = 3) mice. *P < 0.05, **P < 0.01, ***P < 0.001, ((**A**–**G**) Dunn’s Kruskal-Wallis multiple comparisons, (**H**–**I**) Mann-Whitney U test).

Pregnancy increased serum concentrations of progesterone, corticosterone, 11-deoxycorticosterone, aldosterone, 11-dehydrocorticosterone, testosterone, androstenedione and 17α-hydroxyprogesterone (Fig. 5A–H, Supplement Fig. S1F–O). Although a general pattern pointing to a decrease of these hormones by anti-IL-1β appeared, none of these changes were statistically significant. However, in anti-IL-1β treated pregnant oGDM mice the corticosterone precursor 11-deoxycorticosterone numerically increased by 22% (Fig. 5D) while corticosterone (the main glucocorticoid in rodents) itself numerically decreased (Fig. 5F), so that there was a tendency to a reduction of corticosterone/11-deoxycorticosterone ratio (Fig. 5I) compared to vehicle treated pregnant oGDM mice. Supporting this finding, adrenal gene expression of *Cyp11b2*, an enzyme that can convert 11-deoxycorticosterone to corticosterone, was significantly (P < 0.01) increased only in vehicle treated oGDM but not following anti-IL-1β therapy (Fig. 5J), while in oGH mice *Cyp11b2* was not significantly affected by anti-IL-1β antibodies (Fig. 5K). Cyp11b1 that also catalyzes the conversion of 11-deoxycorticosterone to corticosterone was not altered by anti-IL-1β treatment (not shown).

## Discussion

We established a model for gestational diabetes in mice that has the advantage of integrating two main risk factors of the human situation, obesity and aging. This model allowed us to identify IL-1β as a causal driver of impaired glucose tolerance in GDM. Indeed, uterine and placental *Il1b* expression were increased during pregnancy, possibly contributing to the observed increase in circulating IL-1β. Accordingly, IL-1β antagonism improved glycaemia specifically in pregnant oGDM mice and not in non-pregnant controls. This was possibly due to reduced conversion of 11-deoxycorticosterone to corticosterone as a consequence of lower *Cyp11b2* enzyme expression.

Anti-IL-1β was well tolerated in pregnant mice without any apparent influence on maternal and fetal weight or litter size. In humans, two drugs are approved to antagonize the IL-1 system, the anti-IL1β antibody canakinumab and the IL-1 receptor antagonist anakinra. Both have shown high safety profiles in long-term studies with only few serious adverse events during severe infections. Although some data exist about the use of these drugs during human pregnancy^51,52^, clearly possible benefits versus risks would have to be carefully considered.

Anti-IL-1β had no influence on insulin secretion, pointing to an insulin-sensitizing effect rather than an improvement of insulin production in this model. Assessing insulin sensitivity with the gold standard, the hyperinsulinaemic-euglycaemic clamp, involves anesthaesia and surgery. The resulting stress would not be compatible with experimentations during pregnancy precluding us from performing hyperinsulinaemic-euglycaemic-clamp studies in these animals.

We observed increased uterine and placental IL-1β expression during pregnancy as well as upregulation of markers of natural killer cells (*NK1*.*1*), dendritic cells (*Cd74*) and macrophages (*Cd68*, *Adgre*) in oGDM. This is in agreement with a previous report of increased immune-cell infiltration in the uterus during pregnancy^53^. Macrophage markers (*Cd68*, *Adgre*) were also upregulated in uterine tissues of oGH pregnant mice compared to non-pregnant controls. Further, HFD-fed pregnant *Il1b*^fl/fl^ *Lyz2*-Cre knock out mice, which are deficient in myeloid derived IL-1β, did not have improved glucose tolerance compared to pregnant wild type mice. These findings suggest that natural killer cells or dendritic cells rather than macrophages are a likely cellular source of increased uterine IL-1β in GDM. The precise implications of this inflammatory process remain to be shown, but it is likely to contribute to uterus growth and possibly to the fetal-maternal immune barrier. In response to metabolic stress induced by overfeeding and obesity, these immune cells may become pathologically over-activated^54^, leading to secretion of cytokines such as IL-1β, releavant for glucose homeostasis.

We conclude, that IL-1β, presumably derived from the uterus and the placenta, contribute to the impaired glucose tolerance during GDM, possibly via changes in steroid-hormones and subsequent insulin resistance.

## Supplementary information

Supplementary informations.

## Data Availability

The data that support the findings of this study are available from the corresponding author upon request.
