# Supplementary material for: Inhibition of IL-1beta improves Glycaemia in a Mouse Model for Gestational Diabetes
Source: Sci Rep. 2020 Feb 20;10:3035. doi: 10.1038/s41598-020-59701-0 (PMC7033251; doi:10.1038/s41598-020-59701-0)
Supplement: Supplementary file 1 — Supplementary informations. [file 41598_2020_59701_MOESM1_ESM.pdf]

Inhibition of IL-1beta improves Glycaemia in a Mouse Model for Gestational Diabetes

Names and affiliations during the study:

Friederike Schulze*[1], Josua Wehner*[1], Denise V. Kratschmar[2], Valmir Makshana[1], Daniel T. Meier[1], Stéphanie P. Häuselmann[1], Elise Dalmas[1], Constanze Thienel[1], Erez Dror[1], Sophia J. Wiedemann[1], Shuyang Traub[1], Thierry M. Nordmann[1], Leila Rachid[1], Axel De Baat[1], Theresa V Rohm[1], Cheng Zhao[1], Alex Odermatt[2], Marianne Böni-Schnetzler[1], Marc Y. Donath[1]

*equal contribution

[1]Clinic of Endocrinology, Diabetes and Metabolism, University Hospital Basel, Basel, Switzerland and Department of Biomedicine, University of Basel, Basel, Switzerland.
[2]Division of Molecular and Systems Toxicology, Department of Pharmaceutical Sciences, University of Basel, Basel, Switzerland.

Correspondance
Friederike Schulze
Department of Biomedicine
University of Basel
Basel Switzerland
Tel +41798541625
schulze.fritzi@gmail.com

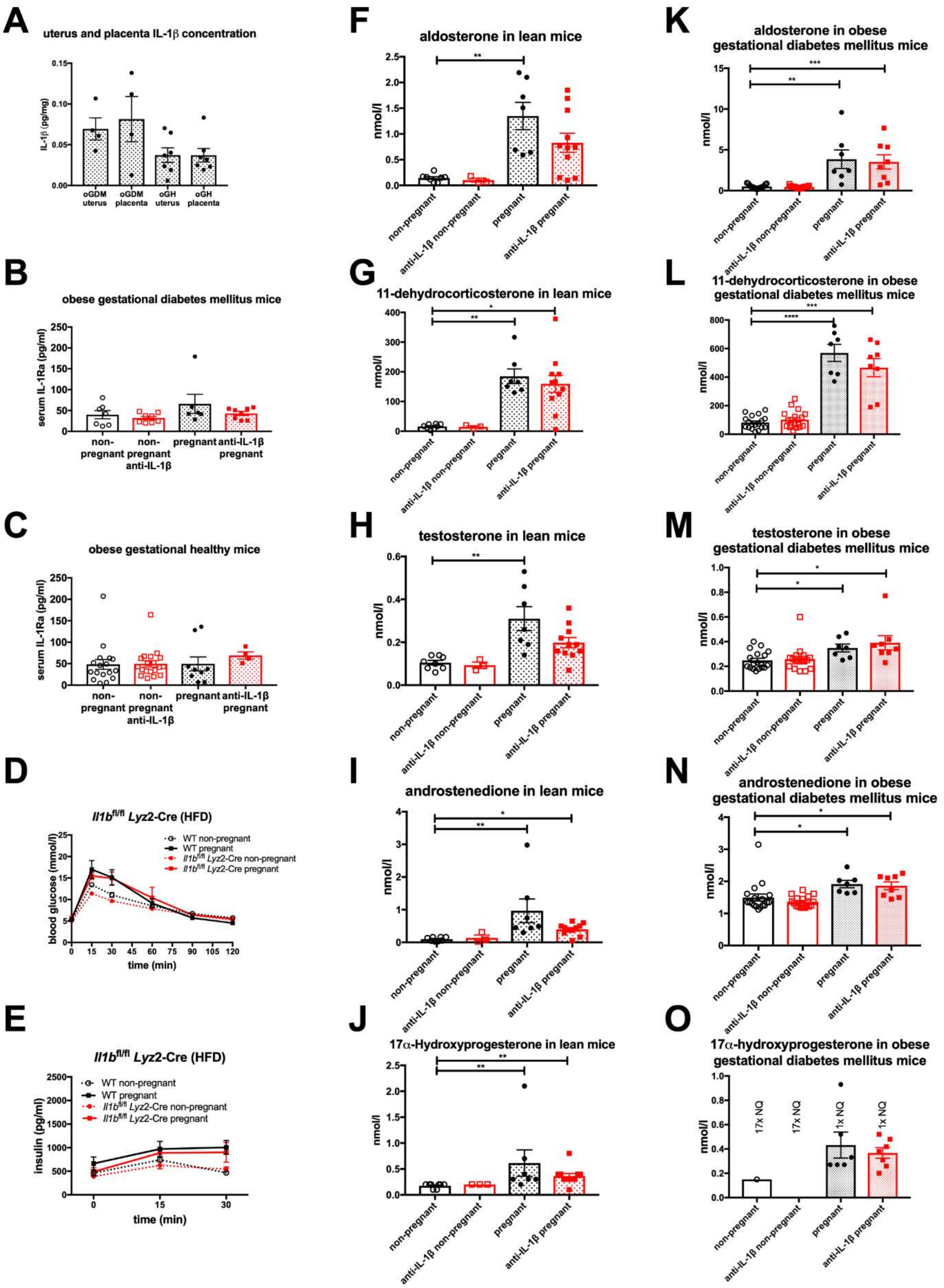

A
uterus and placenta IL-1β concentration
IL-1β (pg/mg)
0.15
0.10
0.05
0.00
oGDM uterus
oGDM placenta
oGH uterus
oGH placenta
B
obese gestational diabetes mellitus mice
serum IL-1Ra (pg/ml)
250
200
150
100
50
0
non-pregnant
non-pregnant anti-IL-1β
pregnant
anti-IL-1β pregnant
C
obese gestational healthy mice
serum IL-1Ra (pg/ml)
non-pregnant
non-pregnant anti-IL-1β
pregnant
anti-IL-1β pregnant
D
Il1b^fl/fl Lyz2-Cre (HFD)
blood glucose (mmol/l)
time (min)
WT non-pregnant
WT pregnant
Il1b^fl/fl Lyz2-Cre non-pregnant
Il1b^fl/fl Lyz2-Cre pregnant
E
Il1b^fl/fl Lyz2-Cre (HFD)
insulin (pg/ml)
time (min)
WT non-pregnant
WT pregnant
Il1b^fl/fl Lyz2-Cre non-pregnant
Il1b^fl/fl Lyz2-Cre pregnant
F
aldosterone in lean mice
G
11-dehydrocorticosterone in lean mice
H
testosterone in lean mice
I
androstenedione in lean mice
J
17α-Hydroxyprogesterone in lean mice
K
aldosterone in obese gestational diabetes mellitus mice
L
11-dehydrocorticosterone in obese gestational diabetes mellitus mice
M
testosterone in obese gestational diabetes mellitus mice
N
androstenedione in obese gestational diabetes mellitus mice
O
17α-hydroxyprogesterone in obese gestational diabetes mellitus mice
17x NQ
17x NQ
1x NQ
1x NQ
nmol/l
non-pregnant
anti-IL-1β non-pregnant
pregnant
anti-IL-1β pregnant

Supplementary Fig. S1

(A)IL-1β per mg tissue in uterus and placenta of pregnant oGDM (n=4) mice and pregnant oGH (n=7) mice.

Concentration of (B) serum IL-1Ra measured in non-pregnant (n=7), anti-IL-1β treated non-pregnant (n=8), pregnant (n=6) and anti-IL-1β treated pregnant (n=8) oGDM mice.

Concentration of (C) serum IL-1Ra measured in non-pregnant (n=17), anti-IL-1β treated non-pregnant (n=19), pregnant (n=10) and anti-IL-1β treated pregnant (n=4) oGH mice.

Concentration of (D) plasma glucose and (E) insulin during a subcutaneous glucose tolerance test in HFD-fed *Il1b*$^{fl/fl}$ *Lyz2*-Cre (non-pregnant n=20, pregnant n=4) and WT (non-pregnant n=18, pregnant n=6) at an age of 15.5 weeks.

(F) Aldosterone, (G) 11-dehydrocorticosterone, (H) testosterone, (I) androstenedione, (J) 17α-hydroxyprogesterone was measured in serum of non-pregnant (n=8), anti-IL-1β treated non-pregnant (n=3), pregnant (n=7) and anti-IL-1β treated pregnant (n=11) lean mice. (K) Aldosterone, (L) 11-dehydrocorticosterone, (M) testosterone, (N) androstenedione, (O) 17α-hydroxyprogesterone was measured in serum of non-pregnant (n=18), anti-IL-1β treated non-pregnant (n=17), pregnant (n=7) and anti-IL-1β treated pregnant (n=8) oGDM mice. NQ= not quantifiable, *P<0.05, **P<0.01, ***P<0.001, ((F-O) Dunn's Kruskal-Wallis multiple comparisons)
